# Demographics of cattle movements in the United Kingdom

**DOI:** 10.1186/1746-6148-7-31

**Published:** 2011-06-28

**Authors:** Matthew C Vernon

**Affiliations:** 1 School of Life Sciences, University of Warwick, Gibbet Hill Road, Coventry, UK

## Abstract

**Background:**

The United Kingdom (UK) government has been recording the births, deaths, and movements of cattle for the last decade. Despite reservations about the accuracy of these data, they represent a large and valuable body of information about the demographics of the UK cattle herd and its contact structure. In this article, a range of demographic data about UK cattle, and particularly their movements, are presented, as well as yearly trends in the patterns of movements.

**Results:**

A clear seasonal pattern is evident in the number of movements of cattle, as are the reductions in movement volume due to foot and mouth disease outbreaks in 2001 and 2007. The distribution of ages of cattle at their time of death is multimodal, and the impact of the over thirty months rule is marked. Most movements occur between agricultural holdings, markets, and slaughterhouses, and there is a non-random pattern to the types of holdings movements occur between. Most animals move only a short distance and a few times in their life. Most movements between any given pair of holdings only occurred once in the last 10 years, but about a third occurred between 2 and 10 times in that period. There is no clear trend to movement patterns in the UK since 2002.

**Conclusions:**

Despite a substantial number of regulatory interventions during the last decade, movement patterns show no clear trend since 2002. The observed patterns in the repeatability of movements, the types of holdings involved in movements, the distances and frequencies of cattle movements, and the batch sizes involved give an insight into the structure of the UK cattle industry, and could act as the basis for a predictive model of livestock movements in the UK.

## Background

### Livestock identification and tracing legislation

The movement of animals within the United Kingdom (UK) is vital to the economics of the livestock industry, but carries with it the risk of transmitting infectious diseases across substantial geographic distances [1-6]. Over the last sixty years, the UK government has introduced increasingly detailed legislation relating to the identification and tracing of cattle. A requirement to identify cattle was first introduced in 1953, as part of the effort to eradicate bovine tuberculosis (BTB). In 1960, the Movement of Animals (Records) Order 1960 (made under the Diseases of Animals Act 1950) required farmers to keep a record of all movements of bovines on or off their premises, and to store these records for three years [7]. In 1990, in response to concerns over bovine spongiform encephalopathy (BSE), tighter controls were introduced. The Bovine Animals (Identification, Marking and Breeding Records) Order 1990 required farmers to record the births of all calves and the identity of their dam, and to keep those records for ten years. Dairy cattle were required to be marked and recorded within 36 hours of birth, and other cattle within 7 days. The Movement of Animals (Records) Amendment Order 1990 extended the period for which movement records had to be kept to 10 years.

The European Economic Community issued Council Directive 92/102/EEC in 1992, which required (amongst other things) movements of cattle to be recorded including origin and destination of the cattle concerned; cattle also had to be identified with an ear tag bearing a code of no more than 14 characters. In the UK, this was implemented by the Bovine Animals (Records, Identification and Movement) Order 1995. That order also required cattle farmers to register their holding with their local Animal Health Office, and introduced the Ear Tag Allocation System to ensure that every bovine animal had a unique identity. In 1996, the Ministry of Agriculture, Fisheries, and Food (MAFF) considered that implementing a computerised Cattle Traceability System (CTS) was necessary to enable the lifting of the export ban on British beef [7]. Accordingly, the CTS was established in September 1998. During the autumn of 2000, the "Cattle Count 2000" exercise was carried out, to register cattle born or imported before the first of July 1996 (when passports were first issued), and to confirm the location of cattle born between then and the twenty-seventh of September 1998 (when the CTS went live). Cattle passports issued since 28 September 1998 take the form of chequebook-style passports (DEFRA form CPP13). These consist of: a front page with details of the animal's eartag, breed, date of birth, and genetic dam, as well as the passport's issue (and, possibly, re-issue) date; a short summary of previous holdings the animal has been on prior to the passport being (re-)issued; movement summary pages into which details of movements of the animal are entered; detachable movement cards by which movements may be reported to the CTS; and a back cover for reporting the animal's death. As of January 2001, it has been a legal requirement to report all movements of bovine animals to the CTS. The British Cattle Movement Service (BCMS) is responsible for running the CTS.

The Rapid Analysis and Detection of Animal-related Risks project (RADAR) was started in 2005 by the Department for Environment Food and Rural Affairs (DEFRA) to collect veterinary surveillance data from different sources in the UK. It is being developed and released in phases between 2005 and 2013. Phase 1 took place in March 2005, and contained information on the UK cattle population as well as data on *Salmonella *cases. The cattle movement data contained within RADAR are supplied by the BCMS [8]. Cattle movements are reported to the BCMS by the holdings at both ends of the movement: i.e. an "off" record is created at one holding, and an "on" record at the other. Part of RADAR phase 1 has been to turn unpaired movements into a life history for each animal. First, duplicate movement records are discarded, as are movements before the birth date, or after the death date (these latter two are presumably due to errors in data entry, either by the farmer, or by BCMS staff). A record of the animal's life history is then generated, consisting of a series of stays at locations (potentially including the "unknown" location), as can best be described by the extant movement records [9]. These movement data have been used as the basis for a broad range of epidemiological models [3,10-13], as well as a smaller body of work on the demographics of the UK cattle industry [14-16].

### Movement control legislation

Movements of bovines since 2001 have not occurred in an unchanging regulatory environment. There have been movement restrictions in the face of specific disease outbreaks: nationwide during the 2001 foot and mouth disease epidemic and more locally during the smaller 2007 epidemic; and from September 2007 onwards to tackle bluetongue. Additionally, regulations have been introduced to try and make the UK cattle herd less susceptible to disease transmission. A six-day standstill period was introduced on 1 August 2003 by the Disease Control (England) Order 2003; this meant that if any sheep, goats, cattle or pigs were moved onto a farm, then no sheep, goats, or cattle could be moved off that farm for 6 days. As an attempt to control the spread of BTB, pre-movement testing of bovines was introduced in a phased manner by the Tuberculosis (England) Order 2006, the Tuberculosis (England) Order 2007, the Tuberculosis (Scotland) Order 2007, and the Tuberculosis (Wales) Order 2006. Bovines on a farm with a 1- or 2-year BTB testing interval in England and Wales being moved must have been tested for BTB within 60 days. In Scotland, animals must additionally be tested 60-120 days post-movement.

### Previous work

Some other previous work on related questions based on RADAR data has been published. DEFRA's Farming Statistics team have published several "Cattle books" containing descriptive statistics on the size, location, breed make-up, and so on of the UK cattle herd. The most recent of these described the cattle herd in 2008, with population statistics such as number and ages of cattle, their breeds and geographic distribution captured as at 1 June 2008 (when the annual June Survey of Agriculture takes place) [17]. Statistical analyses of BCMS movement data have highlighted biases in the reporting of birth dates [16], and the fact that certain classes of movements (specifically, those of older animals, longer-distance movements, and movements to slaughterhouses) are under-reported [18]. Two seasonal peaks in movement volume are observed in the spring and autumn, and most movements of livestock occur during the working week, with a peak on Wednesdays [15,16]. While most animals only move a short distance, there are a small number of animals that move much further. Mitchell and colleagues described the mean distance moved as 58 km, and the maximum as 1000 km [15], while Christley and colleagues considered February 2002, and found the median movement distance to be 39 km, and the maximum 1000 km [14].

The requirement to record the birth, death, and movements of cattle exists across the EU; various member states' data have been employed by researchers interested in agricultural economics or epidemiology. For example, in 2005, most cattle moved within Portugal were young beef stock, and most of the cattle were found in the north of the country; movements in the south of the country were less frequent, but tended to be substantially larger [19]. Natale and colleagues constructed a static network model of the Italian cattle herd based on movements recorded in 2007, as well as considering the types of holdings that participated in movements, and the distances of movements. They used disease simulation to suggest that targetting movement restrictions to affect central (in the network theoretical sense) farms could be a useful disease control tool in the event of an outbreak [20]. In Sweden, Nöremark and colleagues have considered data on cattle and pigs together; similarly to the UK, they found peaks in cattle movements in the spring and autumn, reporting bias in dates, and that most movements of cattle and pigs occurred over a relatively short distance, with a few much longer movements [21]; they went on to perform network analyses of these movement data, and considered the types of holding that were of greatest epidemiological significance within the network [22].

### This article

In this article, RADAR data for the past decade are used to illustrate the demographics of UK cattle, and to consider trends in movement patterns across that decade. This long-term approach is in contrast to previous authors who have typically concentrated on a single year, or a few years at most. The distribution of number of movements, their timings, and the types of holding involved in movement cattle are analysed, as well as the number of cattle in the UK, and the ages and locations at which they die. Since livestock movements are important for the spread of infectious diseases, the frequency and distance cattle move are analysed, as well as how frequently contacts between farms recur.

## Results

### Movement numbers, timing, and holding types

**Figure 1:**
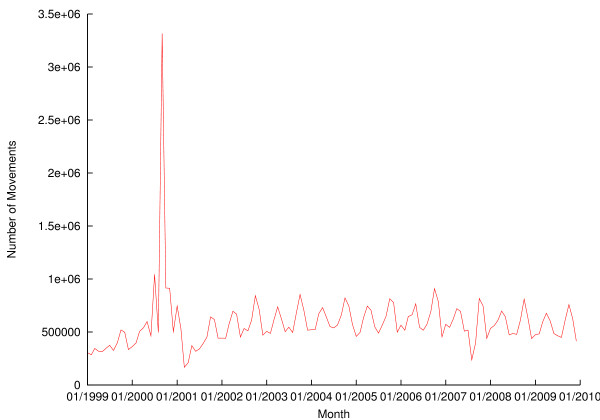
**Numbers of movements of cattle per month for 1999-2009**.

The extract from RADAR supplied by DEFRA contained movements of bovines in the UK up to 14 April 2010. The RADAR livestock movements table contains 157,066,010 records, representing the movements of 43,499,171 distinct animals between 138,640 distinct locations. The number of movements in each month of 1999 to 2009 is plotted in Figure 1. The large spike in the autumn of 2000 is an artifact of Cattle Count 2000, when previously unregistered cattle were registered, and movements from their birth locations to their then-current ones inferred. The quality of pre-2001 movement data remains questionable, however. The number of movements beginning and leaving premises of different types in 2008 and 2009 is shown in table 1; note that births and deaths (where an animal does not move between two holdings) will not appear in these figures.

**Table 1:** Movements from 2008 and 2009, classified by location type.

Abbreviation	Location type	Count	Movements From	Movements To
AH	Agricultural Holding	263,882	11,818,325	7,294,890
AI	AI Sub Centre	45	50	55
CA	Calf Collection Centre	632	28,102	37,829
CC	Collection Centre BSE material	52	35,336	48,256
EX	Export Assembly Centre	76	59,418	65,553
HK	Hunt Kennel	369	316	92
KY	Knackers Yard	140	265	456
LK	Landless Keeper	4,534	196,830	196,165
MA	Market	617	3,251,527	3,754,433
SG	Showground	766	58,032	58,251
SM	Slaughterhouse MP & Cold Store	58	3	4
SR	Slaughterhouse (Red Meat)	1,164	174,601	4,132,026
XX	[Field Left Blank]	87,980	6,151	40,946

"Count" indicates the number of holdings of that type.

Table 2 is a similar table, but the type of holding at both ends of each movement is considered. Expected values (assuming random movements) for the cells of this table may be calculated given the total number of movements for each holding type. These are shown, rounded to the nearest integer, in Table 3; where the observed number of movements was higher than the expected number, the number is in *italic type*, and where the observed number of movements was less than the expected number, the number is in **bold type**. Considering Table 2 as a contingency table, the G statistic [23] is 3225693, with 144 degrees of freedom; the p-value is less than 2.2 × 10 ^-^^16^, showing that there is a statistically significant association between the source and destination holding types.

**Table 2:** Numbers of movements between holdings of different types in 2008 and 2009.

Destination holding type													
	**AH**	**AI**	**CA**	**CC**	**EX**	**HK**	**KY**	**LK**	**MA**	**SG**	**SM**	**SR**	**XX**

AH	4,482,142	52	31,993	42,408	58,357	45	411	117,088	3,563,642	55,812	0	3,432,048	34,327

AI	41	0	0	0	0	0	0	3	1	2	0	3	0

CA	17,077	0	6	64	3	0	0	366	117	0	0	10,442	27

CC	11,010	0	0	0	239	0	0	95	460	0	0	23,477	55

EX	49,096	0	30	0	116	0	0	179	588	9	0	8,997	403

HK	186	0	0	0	0	0	0	0	81	0	0	48	1

KY	155	0	0	0	0	0	3	0	52	0	0	51	4

LK	85,645	3	189	66	82	0	1	4,572	45,998	1,923	0	57,485	866

MA	2,584,531	0	93	2,231	6,304	47	40	71,433	2,429	44	0	579,804	4,571

SG	55,112	0	0	0	32	0	0	1,872	46	425	0	512	33

SM	0	0	0	0	0	0	0	0	0	0	0	3	0

SR	6,753	0	5,506	3,481	405	0	1	436	139,631	17	4	17,749	618

XX	3,142	0	12	6	15	0	0	121	1,388	19	0	1,407	41

The first column contains the source holding type. Holding type abbreviations are defined in table 1.

**Table 3:** Expected numbers of movements between holdings of different types in 2008 and 2009.

Destination holding type													
	**AH**	**AI**	**CA**	**CC**	**EX**	**HK**	**KY**	**LK**	**MA**	**SG**	**SM**	**SR**	**XX**

AH	**5,516,259**	*41*	*28,605*	*36,490*	*49,569*	**69**	*344*	**148,336**	*2,839,032*	*44,048*	**3**	*3,124,560*	*30,962*

AI	*23*	0	0	0	0	0	0	*0*	**12**	*0*	0	**13**	0

CA	*13,116*	0	**68**	**86**	**117**	0	0	*352*	**6,750**	**104**	0	*7,429*	**73**

CC	**16,493**	0	**85**	**109**	*148*	0	**1**	**443**	**8,488**	**131**	0	*9,342*	**92**

EX	*27,733*	0	**143**	**183**	**249**	0	**1**	**745**	**14,273**	**221**	0	**15,709**	*155*

HK	*147*	0	0	0	**1**	0	0	**3**	*75*	**1**	0	**83**	0

KY	*123*	0	0	0	**1**	0	*0*	**3**	**63**	0	0	**70**	*0*

LK	**91,871**	*0*	**476**	**607**	**825**	**1**	**5**	*2,470*	**47,283**	*733*	0	*52,038*	*515*

MA	*1,517,665*	**11**	**7,870**	**10,039**	**13,637**	*19*	**94**	*40,811*	**781,091**	**12,118**	0	**859,647**	**8,518**

SG	*27,086*	0	**140**	**179**	**243**	0	**1**	*728*	**13,940**	*216*	0	**15,342**	**152**

SM	**1**	0	0	0	0	0	0	0	0	0	0	*0*	0

SR	**81,495**	0	*422*	*539*	**732**	**1**	**5**	**2,191**	*41,943*	**650**	*0*	**46,161**	*457*

XX	*2,871*	0	**14**	**18**	**25**	0	0	*77*	**1,477**	**22**	0	**1,626**	*16*

*Italic numbers *show where the observed number was greater than the expected number, **bold numbers **show where the observed number was less than the expected number, and numbers in normal type show where the observed number was within 1 movement of the expected number The first column contains the source holding type. Holding type abbreviations are defined in table 1.

### Livestock numbers and ages

**Figure 2:**
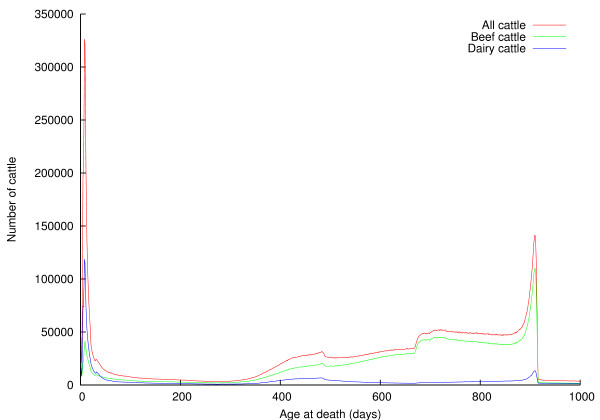
**Distributions of ages of cattle at time of death**.

**Figure 3:**
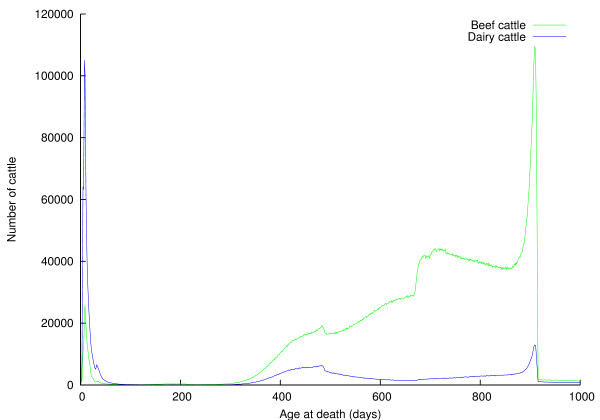
**Distributions of ages of cattle dying on holdings of type "SR" (red meat slaughterhouse)**.

**Figure 4:**
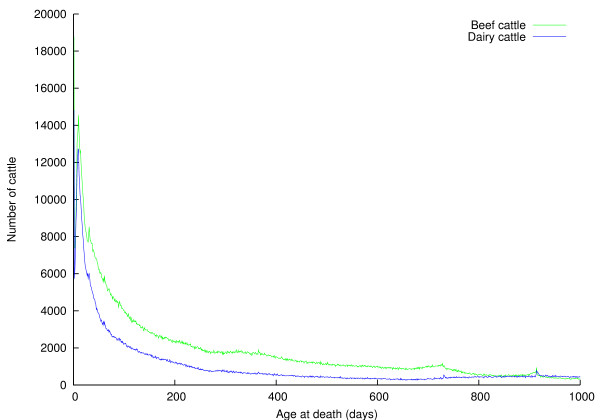
**Distributions of ages of cattle dying on holdings of type "AH" (animal holding)**.

RADAR contains data on 43,499,850 animals (this figure is slightly larger than that quoted above, because some animals have no movement records associated with them), of which 9,088,363 have a birth date but no death date, giving an upper bound on the number of cattle alive in the UK at the time the data were provided. The ages at which cattle die are shown in Figure 2; the peaks are at 8 days, around 16 months, around 24 months, and around 30 months. Table 4 shows the number of animals that died on each holding type; 99% of deaths occur on animal holdings or at red meat slaughterhouses. Figures 3 and 4 show the ages at which cattle die on red meat slaughterhouses and animal holdings, respectively.

**Table 4:** Deaths of cattle, by location type.

Abbreviation	Location type	Deaths
SR	Slaughterhouse (Red Meat)	28,195,725

AH	Agricultural Holding	5,774,117

XX	[Field Left Blank]	198,592

SM	Slaughterhouse MP & Cold Store	130,063

LK	Landless Keeper	41,506

HK	Hunt Kennel	30,634

KY	Knackers Yard	25,527

MA	Market	6,954

CA	Calf Collection Centre	2,467

EX	Export Assembly Centre	798

CC	Collection Centre BSE material	758

HB	Head Boning Plant	287

IN	Incinerator	160

AI	AI Sub Centre	66

SW	Slaughterhouse (White Meat)	43

SG	Showground	23

PP	Protein Processing Plant	9

CR	Cutting Room	4

ET	Embryo Transfer Unit	3

MP	Meat Products Plant	2

### Frequency and distance of movements

**Figure 5:**
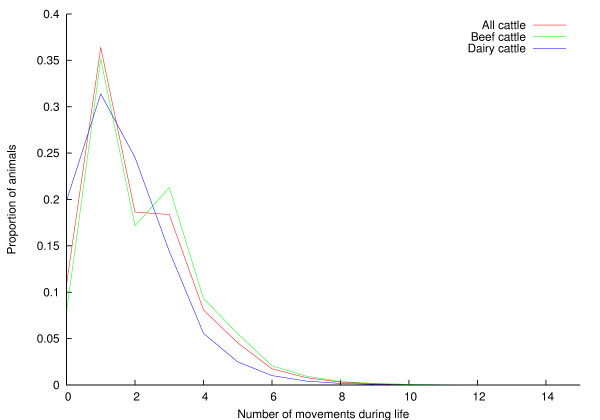
**Distribution of number of moves an animal makes in its life**.

**Figure 6:**
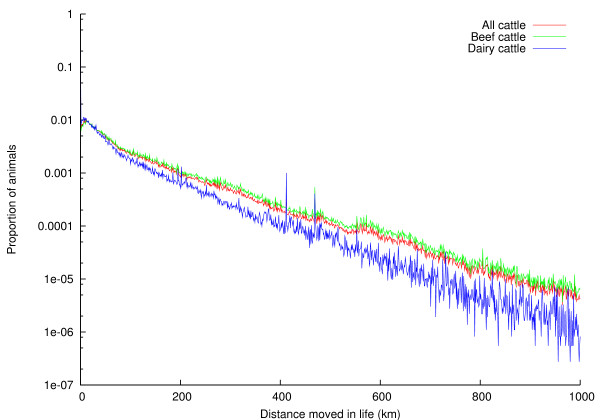
**Distribution of distance an animal moves in its life**.

The distribution of number of times an animal moves in its life is shown in Figure 5. The *x*-axis has been truncated at 15; the largest number of moves in a lifetime according to RADAR is 149. The distributions are shown for all cattle, as well as beef and dairy cattle. The distribution of distances animals move in their life is shown, using a log scale, in Figure 6, again subdivided into beef and dairy cattle. The *x*-axis has been truncated at 1,000 km; the greatest distance moved in the life of a single animal according to RADAR is 4,838 km.

**Figure 7:**
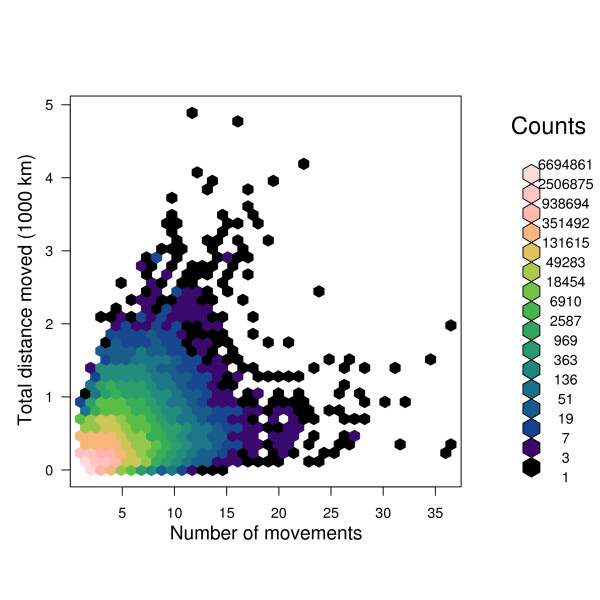
**Number of movements in an animal's life, versus the total distance moved in that animal's life**.

**Figure 8:**
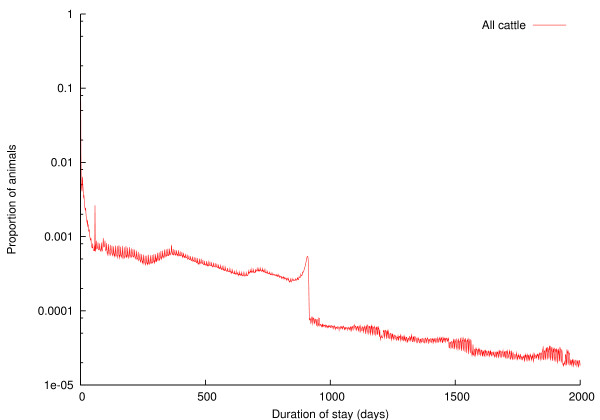
**Distribution of time animals spend on holdings**.

The relationship between the number of times an animal moved in its life and the total distance it moved in its life is examined in Figure 7; coloured hexagonal binning has been used to illustrate the density of animals across the figure [24]. Spearman's rank correlation coefficient *ρ *= 0.496, *p *< 2.2 × 10^-16^, showing a weak but statistically significant correlation between distance moved in life and number of movements in life. The distribution of length of time animals spend on a particular holding is shown in Figure 8. The *x*-axis is truncated at 2000 days (about five and a half years), and the *y*-axis is logarithmic; the longest stay of an animal on a location according to RADAR was 9425 days (around 26 years). The peaks are at around 2 months, and around 30 months.

**Figure 9:**
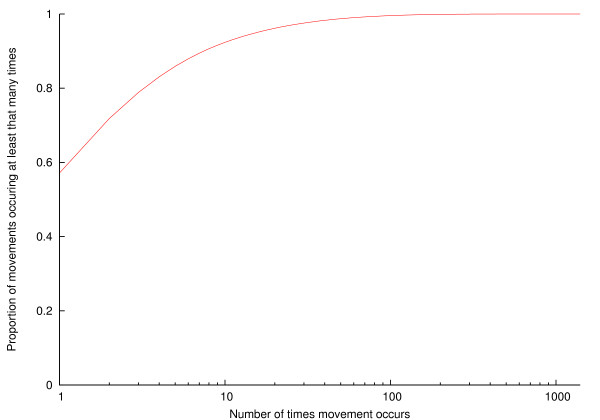
**Cumulative distribution of number of times a movement occurs**.

Figure 9 shows the number of times a movement occurs (i.e. the same source and destination holdings, on different dates) as a cumulative frequency distribution, with a logarithmic *x*-axis. Whilst nearly 60% of movements occur only once, a further 30% occur between 2 and 10 times.

### Changes in movement patterns over the past decade

**Figure 10:**
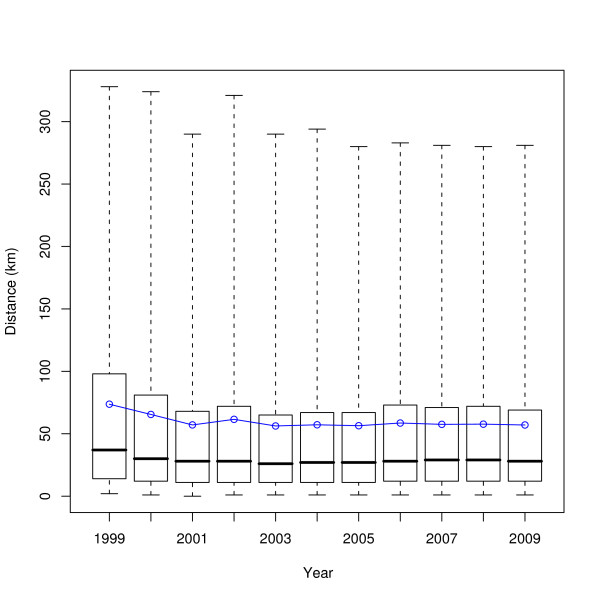
**Change in distances animals are moved over time**. Boxes show the median and quartiles, whiskers show the central ninety-five percentiles, the blue line shows the mean.

**Figure 11:**
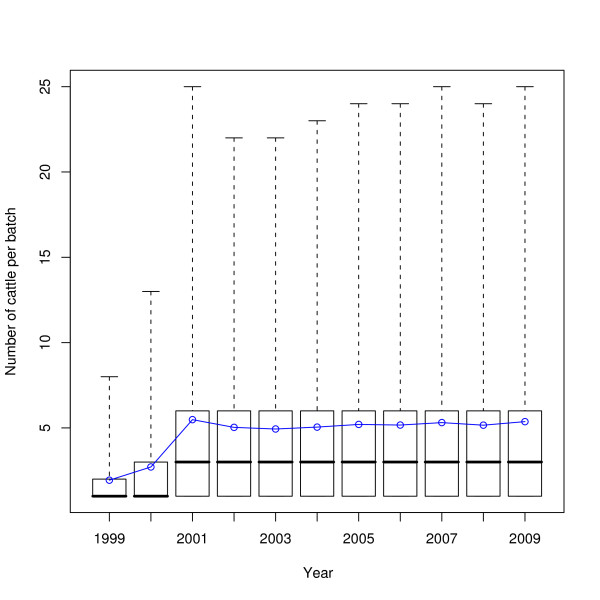
**Change in movement batch sizes over time**. Boxes show the median and quartiles, whiskers show the central ninety-five percentiles, the blue line shows the mean.

**Figure 12:**
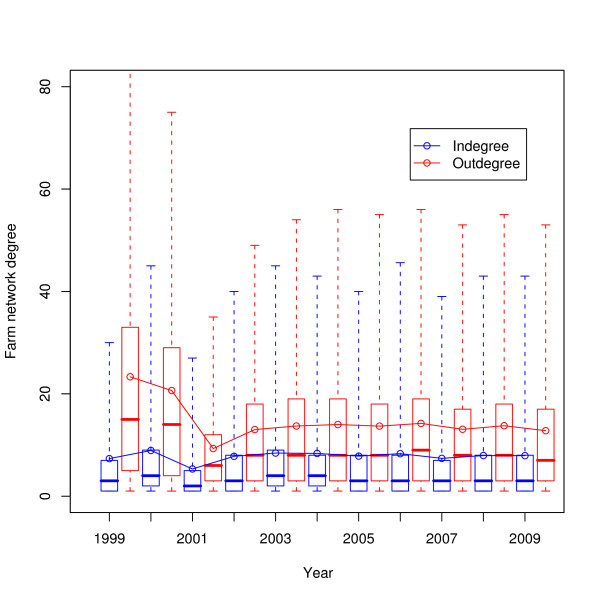
**In- and out-degrees of farms per year**. Boxes show the median and quartiles, whiskers show the central ninety-five percentiles, the solid lines show the mean.

**Figure 13:**
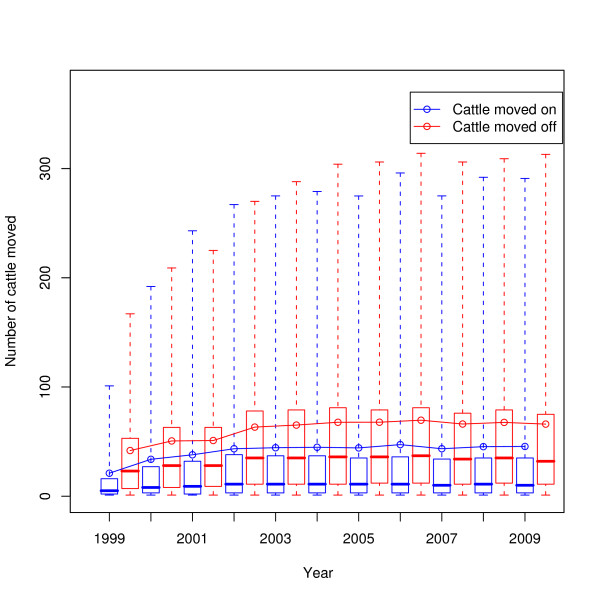
**Numbers of animals moved on and off farms per year**. Boxes show the median and quartiles, whiskers show the central ninety-five percentiles, the solid lines show the mean.

The distances of movements across the years 1999-2009 are shown in Figure 10; the box and whisker plots show the median and interquartiles (box) and the central ninety-five percentiles (whiskers), whilst the blue line indicates the mean. The change in movement batch sizes across the same time period is shown in Figure 11. The in- and out-degrees of farms taking a single static network for each year are shown in Figure 12. The number of cattle moved onto and off farms in a year is shown in Figure 13.

## Discussion

### Movement numbers, timing, and holding types

The foot and mouth disease epidemics in 2001 and 2007 are both noticeable as a drop in movement volume in Figure 1. Even at the height of the 2001 epidemic, however, there was still a certain amount of movement going on; licenses were granted for movements within the infected area, from the uninfected area to the infected area, and within the uninfected area.

Figure 1 shows a clear seasonal pattern to movement volumes, with peaks in April and October of each year. Previous work has looked at seasonal patterns in cattle movements in more detail, and shown both that most movements occur during the working week, with a peak on Wednesdays, and that there is a seasonal peak in the number of births in spring, and a smaller one in September [15,16]; the longer-term analysis presented here shows that this seasonal pattern has continued.

As would be expected, the vast majority of movements involve agricultural holdings, markets, and slaughterhouses. Table 1 shows that agricultural holdings are net exporters of animals, the numbers of animals entering and leaving markets are roughly the same, and that slaughterhouses are net importers of animals. Since animals are born on farms, pass through markets, and die at slaughterhouses, these figures are reassuringly predictable.

Comparing tables 2 and 3 shows that there were substantially fewer movements between animal holdings in 2008-2009 than would be expected by chance; the majority of this difference is explained by the greater number of movements from animal holdings to markets and slaughterhouses, and from markets to animal holdings. Similarly, there is very little movement of animals from market to market, animals instead moving to or from animal holdings. As well as being an interesting insight into the structure of the cattle industry in the UK, these figures would be valuable for constructing an economic model of livestock movements, which in turn might be a useful technique for predicting future patterns of livestock movement in the UK.

### Livestock numbers and ages

Figures 2, 3, and 4, and Table 4 provide some insight into the mortality of British cattle. Table 4 shows unremarkably that the majority of cattle deaths occur at red meat slaughterhouses; also that animal holdings and red meat slaughterhouses account for nearly all (99%) cattle deaths between them. In the light of concerns about the risk BSE posed to human health, The Fresh Meat (Beef Controls) Regulations 1996 were introduced on 29 March 1996. They banned cattle that were over thirty months old from entering the human food chain; instead the animals were slaughtered, and farmers paid compensation under the over thirty months slaughter scheme. This ban was relaxed on 7 November 2005, when older cattle were again eligible to enter the human food chain, provided they tested negative for BSE. The effect of this so-called "over thirty month rule" (OTM) is clear to see in Figures 2 and 3 -- there is a substantial spike in the number of cattle dying at thirty months old. Figure 3 shows the distribution of ages of animals dying at red meat slaughterhouses. There is a substantial peak at around a week of age, particularly among dairy cattle; male dairy calves are worth very little, so some are slaughtered at a young age to save the cost of rearing them; rennet may also be extracted from the abomasums of calves. Animals are typically slaughtered for veal at around 6 months of age; it is clear from Figure 3 that this remains a insignificant beef product in the UK. Intensively reared beef is produced from beef and dairy animals of around 18 months of age; these animals are fed cereals and concentrates and so come to slaughter weight faster than more extensively-reared animals, and the peaks in Figure 3 at around 500 days are due to this type of beef production. Finally, extensive beef suckler systems where beef cattle are reared more slowly on grass result in animals reaching slaughter weight at around 24 months; they result in the step in the number of beef cattle dying at around 700 days old. While Figure 3 shows the relative importance of different beef rearing regimes, Figure 4 shows the ages at which animals die on farms, generally representing a loss to the farmer. As would be expected, the majority of losses occur in young animals, succumbing to disease early in their life, although there is a small peak at 30 months, again probably due to the OTM scheme.

### Frequency and distance of movements

Figures 5 and 6 show the distributions of the number of times an animal moves in its life, and the distances over which animals are moved, respectively. The extreme *x*-values in these figures should be treated with some caution -- it seems unlikely that an animal would travel 4,838 km (roughly four times the road distance between Land's End and John o'Groats) in its lifetime, for example, although pedigree animals may be taken to many showgrounds during their lives. Figure 5 shows that most animals move only a few times during their lifetimes; a single move (from birth location to slaughterhouse) is most common. Dairy animals are more likely to make two moves during their lifetimes than beef animals; this is most likely due to male dairy calves moving once to a fattening unit, and thence to slaughter. Figure 6 shows that while around 18% of animals move less than a kilometre during their life, there is then a very broad spread of distances travelled, with dairy cattle moving less far than beef cattle. This pattern of most animals moving only a few times, and for a short distance has been described before in the UK [15], though the longer-term analysis presented here shows a higher proportion of animals moving only once in their lifetime. Research on cattle movements in Italy, Portugal and Sweden has shown that most movements are short-range (with a few longer distance movements), suggesting that this may be a common pattern across at least Western Europe [19-21]. This is the first analysis to consider the total distance animals move in their lifetimes and its relationship to the number of times animals move.

There is a weak (*ρ *= 0.496) but statistically significant correlation between the number of times an animal is moved in its life, and the total distance it moves, as illustrated in Figure 7. Intuitively, animals that are moved more frequently would be expected to move further in their lifetimes, so it is a little surprising that this correlation is not stronger.

The duration of livestocks' stays on holdings is shown in Figure 8. Around 34% of all recorded stays are transient, i.e. the animal leaves the holding on the same day as it arrived there; these will be stays on markets. The effects of the OTM scheme are evident again, with a noticeable rise in stays of around 30 months.

From the point of view of understanding how cattle are moved, and potentially predicting future movement patterns, an interesting question is how habitual farmers are; if they are very habitual in their movement patterns, then one could reasonably assume that a farm will send its cattle to the same market next year that it did this year. Figure 9 enables this question to be addressed; it shows the number of times a movement occurs as a cumulative frequency distribution. Nearly a third of movements occur between 2 and 10 times, so some repetition of movements should be incorporated into any model of the UK cattle industry, but only to a limited extent.

### Changes in movement patterns over the past decade

Given that the regulatory regime regarding animal movements has changed substantially in the recent past, particularly since the 2001 FMD epidemic, it is worthwhile to try and assess what effect these changes have had on the movement of animals. Figures 10, 11, 12, and 13 do this, on a yearly basis.

What is striking about these figures is how little has changed since 2002 overall, in contrast to work by Robinson and Christley which considered movements in the period 2002 to early 2005 [12]. The availability of data for a longer period of time shows that while there was an increase in cattle movement in the period they studied (see e.g. Figure 13), that increase has not continued.

### Issues with the CTS

The CTS was not set up with the intention that it might be useful as a control system for epidemic diseases such as FMD; the 2001 FMD outbreak in the UK and subsequent enquiries have led to changes in the collection of data, and the scope of such data. Specifically, the UK government has attempted to increase reporting of cattle movements by electronic means, and has introduced schemes to collect details on batch movements (rather than individual-level data) of sheep, pigs, and goats [25].

Not all movements of cattle are required to be reported to RADAR. Specifically, movements to shared grazing lands are not required to be reported, and neither are movements between holdings that have been "linked". The latter process is meant to allow farmers to move livestock between nearby holdings without the administrative burden of having to report the movements, but it has been abused by some farmers, who have "linked" holdings which are far away from each other [25]. Given the original purpose of CTS, it is perhaps unsurprising that such movements need not be reported, but they may represent a substantial epidemiological risk.

A National Audit Office report noted that some keepers may be tempted to avoid the extra work associated with reporting animal movements, and that furthermore there may be financial advantages to deliberately contravening the identification and tracking requirements (particularly given standstill periods); some examples of detected fraud were illustrated, although there is little idea as to the scale of the problem [25]. DEFRA has conducted a review of the livestock movement controls. In addition to issues regarding abuse of "linked" holdings, the review concluded that the current regulations are overly complex and should therefore be simplified. It additionally recommends that abattoirs should report the premises of departure of animals arriving at them, and that markets and collection centres should report the source and destination of animals passing through them, by electronic means. Regarding shared grazing lands, it suggests that a single Land Management Unit should be formed consisting of the common land and any in-bye land to which cattle on the shared grazing have free access; movements into and out of this area would have to be reported, and would induce a standstill period. It also advocates greater regulation of dealers and traders, specifically that those which hold livestock for mixing and sorting purposes be treated as collection centres (and so be subject to a formal approval procedure), and that CTS investigate movements of animals where a few days have passed between an "off" movement and the subsequent "on" movement, to attempt to determine whether the animals concerned stayed at an intermediate premises [26]. In 2010, DEFRA consulted on proposals to simplify the livestock movement rules and holding identifiers in England, although no changes have yet been proposed as a result of that consultation. Problems remain, however. The current regulations are complex, which leads to errors in reporting, and are somewhat open to abuse. Furthermore, the data are not collected nor stored in a manner ideally suited to contact-network-based studies (although this latter situation has improved significantly with the production of ordered movement tables for each animal). How important the delay between movements and their reporting to RADAR is in terms of intervention during an outbreak is an unanswered question; during the brief 2007 FMD outbreak, livestock movement data were not available to researchers until the outbreak was over.

The importance of movements that are not required to be reported to RADAR in contact networks is unknown, and difficult to quantify nationally; a study in the Outer Hebridies showed that shared grazing land was a significant source of unreported contact between different keepers' cattle [27].

## Conclusions

RADAR's data provide an unprecedented opportunity for research into the life, movements, and death of UK cattle. This article has concentrated on the demographics of cattle and their movements. The cattle movement data may also be used to construct a contact network of UK cattle farms; this large-scale network is one of the best-characterised epidemiological networks available, making it a useful tool for research into the relevance of contact networks for epidemiology [28].

There are some apparent similarities between the UK cattle herd's movement patterns and those of other European countries: there are seasonal peaks in movement volumes in Spring and Autumn, and most animals have only a few short movements in their lives, while a few cover much more substantial differences. It would be worthwhile to consider the similarities and differences between different European countries' livestock movements patterns, particularly from the point of view of infectious diseases moving across the continent.

It is interesting to note that despite the significant changes to livestock movement regulations in the last decade, Figures 10, 11, 12, and 13 show that the overall pattern and volume of livestock movements have not changed a great deal since 2002. Despite this, the disease susceptibility of the UK cattle herd (assessed by stochastic simulation) has fluctuated substantially during this period (Vernon and Keeling, in preparation). Therefore, whilst demographic studies such as this are interesting in their own right, care must be taken when employing coarse measures of livestock movements for epidemiological problems.

## Methods

Cattle movement data were provided by DEFRA from the RADAR project on 5 May 2010, containing movements of bovine livestock in the UK until 14 April 2010. In this article, only movements between 1 January 1999 and 31 December 2009 inclusive were considered. Movement data were stored in a Postgresql database instance.

The main information in this database is a "livestock location" table, with each row containing the following information: the identity of the location and animal, the arrival and departure dates, the type of arrival and departure movements (including details of how they were inferred, if relevant), and the country imported from or exported to, if relevant. To derive movements (the edges in a contact network) from this table, it was necessary to find two stays on locations where the animal concerned is the same, and the end date of one stay is the start date of the other; additionally, the start and end locations of the movement should be different, and the movement type by which the animal arrives at the destination holding should not be birth or death. Additionally, there is a "PAF location" table, which contains details of the locations of livestock holdings derived from the postcode address file; this table contains (amongst other things) the eastings and northings of the address associated with a holding. These eastings and northings data were used to calculate the straight-line distances between holdings.

Classification of cattle breeds as beef or dairy follows that used in DEFRA's "Cattle Book 2008" [17]. A static network for each year was constructed by representing each holding that moved any animals in that year as a node, and placing a directed edge between every pair of nodes where there was a movement of cattle between the corresponding nodes in the relevant year. The in-degree of a node is defined as the number of edges that end at that node, and the out-degree of a node is the number of edges that start from that node.

Data handling other than that done using SQL was performed with python scripts, network analyses were performed using Contagion [29], and statistical analyses were performed using R [30].

## Authors' contributions

MCV performed the data analysis, and wrote the paper.
